# Atlanta Academy of Medicine

**Published:** 1871-04

**Authors:** 


### ATLANTA ACADEMY OF MEDICINE.

The old Atlanta Medical Society having expired, the profession in this city have had no organization to regulate, control, or protect their interests, and finding an indispensable necessity for such a standard of authority, they have, with great unanimity, organized the Atlanta Academy of Medicine, upon the very highest principles of the profession, and upon the broadest base with reference to the advancement of medical science.

It embraces all the leading physicians and nearly the whole of the entire body of the medical men, and aims at nothing less than *taking charge* of the interests of the profession in this city, in regulating, controlling, and protecting its members, as well as guarding the interest of the community against the pretensions of irregular or disorderly parties with whom we have been peculiarly cursed. It specially, however, aims to do something towards the direct advancement of medical science, not only in stimulating its members to individual improvement, but by furnishing to the profession, from time to time, through the journals of the country, and through a volume of transactions, the result of its labors. To this end, *Sections*, or Standing Committees have been organized upon all the various departments of medicine and surgery, viz: Anatomy, Physiology, Chemistry, Materia Medica and Therapeutics, Surgery, Obstetrics and Gynæcology, and Practical Medicine, with others on Ethics, Publication and Arrangements, whose duty it is to report fully to the weekly meetings of the Academy upon such subjects as may be, from time to time, referred to them, besides furnishing regularly, in rotation, questions or subjects for discussion.

The Academy is furnishing itself with the leading journals of this country and Europe, and proposes to establish a Library and Museum, and solicits contributions of pathological specimens, of apparatus or of books from the medical men of the State or of the United States, who may feel an interest in the advancement of the objects of the organization.

From this source we expect, confidently, to draw a large amount of valuable material, in the shape of reports of cases and of the discussions before the body, besides, valuable papers of a more systematic character, which will be presented by individual members and by the Sections; the Corresponding Secretary haviug been authorized, upon our application, to furnish such of either as are likely to be interesting and profitable to our readers and while we do not present ourselves as the organ of this body we are satisfied that the relation between us will be mutually beneficial. By the Constitution, the membership of the Academy is not confined to the city, but embraces honorary and corresponding members, who may be elected at their own instance or that of the Academy, without regard to residence. It is believed that it will gradually number among its members a large portion of the more prominent of the profession throughout the State, who will contribute to its success by an occasional attendance upon its meetings when business or pleassure may bring them to the Capitol, and by contributions to its archives, library and museum.

The official organization for 1871, is as follows:

PRESIDENT:

J. P. LOGAN, M. D.

VICE-PRESIDENTS:

1st. J. F. ALEXANDER, M. D.
2d. J. M. BORING, M. D.,
3d. N. D'ALVIGNY, M. D.,

RECORDING SECRETARY AND TREASURER:

R. Q. STACY, M. D.

CORRESRONDING SECRETARY:

WM. ABRAM LOVE, M. D.

LIBRARIAN:

W. N. JUDSON, M. D.

### ORGANIZED SECTIONS.

---

ANATOMY:

W. S. ARMSTRONG, M. D., Ch'n.

E. P. INGRAHAM, M. D.

S. H. STOUT, M. D.

J. T. JOHNSON, M. D.

J. W. VANCE, M. D.

E. D. WITHERS, M. D.

PHYSIOLOGY.

H. V. M. MILLER, M. D., Ch'n.

WM. ABRAM LOVE, M. D.

JOHN M. JOHNSON, M. D.

GASTON ARMSTRONG, M. D.

W. C. MOORE, M. D.

J. H. LOWE, M. D.

CHEMISTRY.

R. Q. STACY, M. D., Chairman.

J. G. WESTMORELAND, M. D.

J. N. SIMMONS, M. D.

C. A. SIPMSON, M. D.

B. D. REEVES, M. D.

J. W. McFAUL, M. D.

MATERIA MEDICA AND THERAPEUTICS.

J. G. WESTMORELAND, M. D., Chairman.

J. M. BORING, M. D.

J. H. LOWE, M. D.

J. M. JOHNSON, M. D.

W. N. JUDSON, M. D.

G. S. CASSIN, M. D.

SURGERY.

W. F. WESTMORELAND, M. D., Chairman.

L. H. ORME, M. D.

E. D. WITHERS, M. D.

W. M. WELLS, M. D.

N. D'ALVIGNY, M. D.

E. S. RAY, M. D.

OBSTETRICS AND GYNÆCOLOGY.

WM. ABRAM LOVE, M. D., Ch'n.

J. GILBERT, M. D.

J. F. ALEXANDER, M. D.

H. V. M. MILLER, M. D.

J. M. BORING. M. D.

D. C. O'KEEFE, M. D.

PRACTICAL MEDICINE.

J. F. ALEXANDER, M. D. Ch'n

S. H. STOUT, M. D.

D. C. O'KEEFE, M. D.

E. P. INGRAHAM, M. D.

W. P. HARDEN, M. D.

T. R. COOK, M. D.

ETHICS.

S. H. STOUT, M. D., Chairman.

E. D. WITHERS, M. D.

WM. ABRAM LOVE, M. D.

W. M. WELLS, M. D.

J. F. ALEXANDER, M. D.

E. S. RAY, M. D.

CONTSITUTION AND BY-LAWS.

LOUIS H. ORME, M. D., Chairman.

J. F. ALEXANDER, M. D.

J. H. LOWE, M. D.

W. N. JUDSON, M. D.

R. Q. STACY, M. D.

J. G. WESTMORELAND, M. D.

PUBLICATIONS.

President and Recording and Corresponding Secretaries.

ARRANGEMENTS.

WM. ABRAM LOVE, M. D. Ch'n

E. S. RAY, M. D.

W. N. JUDSON, M. D.

L. H. ORME, M. D.

W. P. HARDEN, M. D.

J. W. VANCE, M. D.

